# Environmental and psychosocial correlates of physical activity in pregnant women: a study protocol

**DOI:** 10.3389/fgwh.2025.1450342

**Published:** 2025-06-19

**Authors:** Lourdes Margaix-Fontestad, Javier Molina-García, Begoña Arriero-Hurtado, Julia Asensi-Tarín, Ana Queralt

**Affiliations:** ^1^ Puçol Primary Care Centre, Sagunto Health Department, Sagunto Hospital, Sagunto, Spain; ^2^ Foundation for the Promotion of Health and Biomedical Research in the Valencian Region (FISABIO), Valencia, Spain; ^3^ AFIPS Research Group, Department of Nursing, University of Valencia, Valencia, Spain; ^4^ Epidemiology and Environmental Health Joint Research Unit, FISABIO-UJI-UV, Valencia, Spain; ^5^ AFIPS Research Group, Department of Teaching of Physical Education, Arts and Music, University of Valencia, Valencia, Spain; ^6^ Font de Sant Lluís Primary Care Centre, Dr Peset Health Department, Valencia, Spain

**Keywords:** accelerometry, built environment, geographic information systems, healthy lifestyles, physical activity, pregnancy, psychosocial, women

## Abstract

Pregnant women are often more open to engaging in healthy lifestyles. Numerous studies have shown that physical activity (PA) during pregnancy contributes to obstetric and neonatal benefits. Nonetheless, most pregnant women do not comply with PA recommendations. We will analyze the association between environmental and psychosocial variables and PA levels during pregnancy. This observational and cross-sectional study will include pregnant women in their second trimester. Questionnaires will assess obstetric, psychosocial, built-environmental perceptions, and PA factors. PA will also be objectively measured using accelerometers. Geographic Information Systems will objectively analyze built-environment attributes. Birth and neonatal variables will be obtained from patient clinical histories. Multiple regression models will be used to evaluate how personal, psychosocial, and environmental variables influence PA. This study will potentially contribute to characterize the environments that favor PA during pregnancy.

## 1 Introduction

Pregnancy is a life stage during which women are usually more open to the introduction of healthy lifestyle changes that could promote greater benefits to them and to fetal development (1). Interventions regarding engagement in physical activity (PA) with the aim of promoting the health of pregnant women are common (1). In its last publication (2020), the World Health Organization recommended women complete at least 150 min per week of moderate-intensity aerobic activity during pregnancy (2). Furthermore, many countries including Australia, Canada, Denmark, the United States (US), France, Great Britain, and Norway, have their own guidelines on recommended PA levels during pregnancy which include advice on the best types of PA, the duration, frequency (3, 4). In addition, they also include contraindications for engaging in PA and activities not recommended during pregnancy (2, 3, 5). Regarding contraindications, those are mainly obstetric conditions that require bed rest such as a risk of premature birth (6). Besides, some activities such as those with a high risk of falls, physical contact, or that might limit oxygenation, are not recommended (2). However, different studies carried out in various geographical contexts underscore that only a small proportion of pregnant women meet PA levels (from 10% to 27%) recommended in the guidelines (7–9). If we look specifically at the Spanish population, only 25% of pregnant women met the PA recommendations in a study conducted in Granada (10). As mentioned before, the World Health Organization (2) recommended moderate-intensity PA. However, pregnant women who were physically active and engaged in vigorous intensity aerobic activity before pregnancy, can continue vigorous PA during pregnancy, provided they have no medical or obstetric contraindications (2).

Furthermore, according to the academic literature, PA is a healthy behavior that is influenced by the interaction of different factors (11). Ecological models allow us to understand this idea and establish the foundations for the study of factors (individual, social, environmental, and even political models) associated with PA behavior (11). Research has traditionally focused on analyzing the individual and social determinants of engaging in PA. However, in recent decades, studies focusing on environmental factors, particularly those associated with the built environment and PA levels or the incidence of obesity, have also been published (12, 13). Most of this research has been conducted in healthy adults (13, 14), and even in adolescent and child populations (15, 16).

When analyzing the predictors of PA behavior during pregnancy, St-Laurent et al. found that engaging in PA prior to pregnancy was the factor that most influenced moderate to vigorous PA performance during pregnancy (17). Thus, pregnant women who engaged in exercise or walking activities prior to pregnancy, are more motivated and engaged in higher levels of light to moderate activity during pregnancy and postpartum (18). This was also the case for pregnant women who had more children; likewise, having more cars at home or working long hours was related to more sedentary time among pregnant women (19). Additionally, social support for PA by family members or other pregnant women, can help them to be physically active, because their behavior is strongly influenced by the beliefs and attitudes of their social environment, particularly family and friends (20, 21).

Although studies on pregnancy-related behaviors often include PA, tobacco use, and alcohol consumption (22), research specifically exploring the relationship between PA and tobacco or alcohol consumption remains scarce. This may be due to the absence of a significant association (23) or, more commonly, because these variables have not been analyzed together (22, 24). However, one study indicated that pregnant women who engage in recreational PA have a lower risk of smoking during pregnancy, whereas those with a sedentary lifestyle, exceeding 600 min per day, are at a higher risk of smoking (25).

Pregnant women's PA is associated with maternal benefits such as reducing gestational weight gain, lowering the incidence of gestational diabetes and hypertension (10), shortening the first labor period duration, decreasing the number of gestational weeks in full-term fetuses, and reducing postpartum depressive symptoms (26). In recent years, there has been a growing focus on the assessment of pregnant women's mental health due to its high prevalence, with depression affecting between 15% and 23% of pregnant women and moderate to high anxiety occurring in 14% to 29% of cases (27, 28). In this regard, some studies found relationships between high levels of PA and lower levels of prenatal depression and anxiety in pregnant women (29–32). Moreover, in relation to benefits for the fetus, engaging in PA during pregnancy reduces neonatal adiposity (33), increased oxygenation of the umbilical artery and higher umbilical pH levels, thus making the fetus more resistant to stress (10). In turn, this could explain the lower risk of cesarean section as a result of fetal distress at the time of delivery (10). On the other hand, women with more sedentary lifestyles could present shorter gestational age at birth (34), higher fasting glucose and insulin (35), a higher risk of increased C-reactive protein levels (36), a greater risk of fetal acidosis during childbirth (10), and more neonatal adiposity (35). The mechanisms underlying these outcomes remain unclear. Therefore, further studies are needed to determine the reasons for adverse outcomes.

As mentioned before, another factor that influences PA is the built environment. Walkability is one of the most commonly used concepts in the analysis of the built environment. This term refers to different characteristics of the built environment that are significantly related to active lifestyles (14). The most walkable urban environments are usually those that present, among other aspects (37) a great diversity of land uses (i.e., a variety of possible destinations to travel to, such as leisure or sports facilities); a greater residential density that favors, among other considerations, more social relationships; and high levels of connectivity between its streets, thereby allowing the use of a greater range of possible routes to engage in active movement. According to Kershaw et al., built environment attributes have similar PA-promoting benefits in pregnant women as seen in other populations (38). However, research focuses on the built environment and pregnancy is not comparable to other populations because women's PA usually changes during pregnancy (37, 39). Some recent studies found that a more walkable environment is correlated with higher levels of PA in pregnant women (40–42).

Regarding neighborhood socioeconomic levels, some studies indicate that people from families with lower incomes or who live in more disadvantaged neighborhoods are less likely to engage in PA (43). There is a lack of studies analyzing the role of neighborhood socioeconomic status among pregnant women's PA. Likewise, perceived crime and safety did influence pregnant women's PA (44). Another attribute of the urban environment, which consistently correlates with PA levels in pregnant women (42), youth (45, 46) and general population (14), is the presence and density of parks or green areas (14, 42, 45, 46). Recreational activity is favored in these spaces not only because of the facilities offered in them and their inherent aesthetic qualities, but because they represent a destination people can actively travel to.

To the best of our knowledge, there are few studies conducted in pregnant women that included built environmental factors as potential predictors of PA behavior (38, 40–42, 44). Furthermore, the characteristics of the built environment in those studies, which are mainly from the United States, are very different from those of European countries and so other urban environments must be analyzed to clarify the factors that determine PA levels in each context (13).

Therefore, the main objective of this proposed research will be to examine the associations between different social and built environmental factors, engagement in PA, and other health-related outcomes during pregnancy. The degree of compliance with PA recommendations among pregnant women will also be assessed. The general hypothesis of this proposed work is that pregnant women living in more favorable social and built environments will present higher levels of engagement in PA and better health outcomes. The main specific hypothesis are: (1) Pregnant women who live in a more favorable built environment will have higher engagement in PA; (2) Pregnant women who will score lower on anxiety and depression scales will have higher engagement in PA; (3) Pregnant women who have more family and peer support to PA will have higher engagement in PA; (4) Pregnant women who consume less tobacco and alcohol will have higher engagement in PA; (5) Pregnant women who are sufficiently active will have better maternal and fetus health indicators.

## 2 Methods and analysis

The EnsMovEm (Entorno saludable, Movimiento y Embarazo or Healthy Environment, Movement, and Pregnancy) project will be a multicenter, cross-sectional study designed to explore the relationship between social and environmental factors and the level of PA pregnant women. The study population will comprise pregnant women from different Health Departments (Sagunto, Valencia-Doctor Peset, and Alcoy) in the Valencian Community (Spain). The inclusion criteria will be women aged 16 years or more, and in the second trimester of pregnancy, to avoid barriers described in the literature (47, 48). The exclusion criteria will be difficulties in communicating in Spanish or any contraindication for engaging in PA. All pregnant women who meet the inclusion criteria when they attend the midwife's office will be invited to participate. All the participants will be involved with the work on a voluntary basis and must provide their written, informed consent to participation before any study data are collected.

### 2.1 Variables

#### 2.1.1 Physical activity

PA will be measured with the Pregnancy Infection and Nutrition 3 (PIN-3) questionnaire (49), an instrument specifically validated for pregnant women. The PIN-3 records the frequency, duration, and intensity of PA in each domain (recreational, household, child and adult care, transportation, and work). Furthermore, each participant's history of PA before pregnancy will also be measured using the PIN-3 questionnaire. We will also use Actigraph GT3X + and wGT3X-BT accelerometers (50) to provide an objective measure of PA. Participants will wear the accelerometer for a week and complete an accelerometer diary. The intensity of PA will be classified using the Troiano cut-off points (51): 0–100 (sedentary), 100–2019 (light), 2020–5,998 (moderate), and ≥5,999 (vigorous).

#### 2.1.2 Psychosocial metrics

Anxiety will be measured with the Generalized Anxiety Disorder Scale (GAD-7) and its short version, the GAD-2 (52, 53). The risk of depression will be measured using the Edinburgh Postnatal Depression Scale (EPDS) which has been used successfully in pregnant women (54–57), as well as the Whooley questions (57, 58). The use of a few questions (derived from the Whooley and GAD-2 questionnaires) during pregnancy check-ups might improve risk screening for the mental illnesses that most frequently present during pregnancy. All of these tools have been recommended by the National Institute for Health and Care Excellence (NICE) clinical guidelines (59). If someone scores having or at risk of having depression or anxiety, they will continue in the study but they will be referred to other professionals, if necessary. Family and peer influences will be measured with the social support scale for engaging in PA (60) comprising six items scored on a five-point Likert-type scale ranging from 5 (many times) to 1 (never).

#### 2.1.3 Environmental metrics

Perceived environmental factors will be measured with the ALPHA environmental questionnaire (61). This instrument assesses the characteristics of the neighborhood, defined as “the area ALL around your home that you could walk to in 10–15 min—approx. 1.5 km”. It includes questions concerning the type of residence, distance to local facilities, walking or cycle infrastructure, maintenance of infrastructure, safety, how pleasant the area is, cycling and walking networks, and the home environment, all of them within a neighborhood; it also includes the workplace or study environment. Objective characteristics of the built environment such as the population density, street connectivity, socio-economic level (average income and/or educational level of the neighborhood), and presence of and access to parks and green areas will be analyzed using geographic information systems (GIS). Information sources include Spanish Institute for National Statistics (INE), National Geographic Institute of Spain (IGN) and OpenStreetMap (OSM).

#### 2.1.4 Sociodemographic

The following sociodemographic data will also be recorded as part of the questionnaire: age (years), educational level (incomplete primary education, primary education, secondary education, or university-level education), employment status (employed, on sick leave because of health or safety risk factors related to pregnancy, student, homemaker, unemployed, others), and their home address. The home address will be used to geolocate and calculate the objective characteristics of the built environment of pregnant women.

#### 2.1.5 Other health outcomes

We will also ask the participants about their tobacco and alcohol use while pregnant.

#### 2.1.6 Anthropometrics

The participants' height (m), weight (kg) will be objectively measured which we will use to calculate their body mass index (BMI) as body mass divided by height squared.

#### 2.1.7 Pregnancy, birth and neonatal period

There are many variables related to pregnancy, birth and neonatal period included in this study protocol. Some of them have been retained based on the literature research, as there is evidence of associations with PA. Others could be used as covariables to adjust the data analyses or researchers consider them of interest to explore the possible presence of any potential correlation with PA.

Data on the number of pregnancies, vaginal births or cesarean section births, abortions or miscarriages, last menstrual period, and pregnancy week at the time of completing the questionnaire will be recorded through the questionnaire. Moreover, at the first postpartum visit to the midwife, data about the presentation of gestational diabetes, hypertension, or gestational weight gain will be collected by the midwife.

Baby's date of birth, hospital (public or private), type of birth (natural, assisted, or cesarean section), weeks of gestation at birth, newborn weight, and APGAR test at one and five minutes. These birth and neonatal data will be collected from the electronic medical record of the participant at their first postpartum visit to the midwife.

### 2.2 Participation timeline

Participants will be recruited at their health center through a midwife consultation. All of the participants will then be asked to fill out a self-administered questionnaire that includes most of the variables mentioned above, except objective PA and objective environmental measures. The questionnaire will be administered in paper format and will take approximately 15–20 min to complete. Following this, they will be fitted with accelerometers to be worn for a week from the time they wake up until they go to bed, with the exception of while sleeping or doing water-based activities such as swimming or showering. Written and verbal instructions will be provided to each participant in a folder. After completing monitoring for a week, participants will return the accelerometers to the health center, along with the accelerometer diary, in the folder. Study recruitment started in January 2021.

### 2.3 Sample size

We used a multiple regression model to determine the sample size required to analyze the main study objective of evaluating the relationship between the PA levels (in hours/week) of pregnant women and their personal characteristics, psychosocial variables, and the environment in which they live. Thus, accepting an alpha risk of 0.05 and a beta risk of 0.1, with a mean effect size of f2 = 0.15 in the R2 of the model and with 15 predictors, we calculated that 171 participants will be required for this work. In accordance with a methodological review (62), we decided to increase our initial sample size by 15% to compensate for possible dropouts and losses to follow-up. Thus, we propose the recruitment of an initial sample of 200 pregnant women.

### 2.4 Statistical methods

Firstly, given that the objective variables of the built environment will be calculated for each population, we can start this process before or in parallel to the data collection at the health centers. As mentioned in the previous section, a GIS will be used for this purpose; once the sample is collected, the participants will be geolocated through this system. Secondly, a descriptive analysis of the study variables will be carried out. Qualitative variables will be described by absolute frequencies and percentages, while quantitative variables will be summarized through their means, standard deviations, and quartiles.

Likewise, we will conduct a bivariate analysis to study the relationship between the level of PA completed by pregnant women and their sociodemographic characteristics, obstetric and neonatal results, anxiety/depression levels, socioeconomic level, social support for PA practice, PA history, and characteristics of the environment. Student *t*-tests or ANOVAs will be used to compare the PA levels of the participants according to the categories of the qualitative variables; Pearson correlation tests will be used to study the relationships between the quantitative variables. If the applicability conditions of these tests are not verified, non-parametric tests will be used.

To evaluate how the personal, social characteristics, and environment in which each pregnant woman lives influence her performance of PA, we will build a multiple regression model. We also plan to explore the relationship between variables using self-organizing maps (SOM) and/or structural equation modelling.

The dependent variable of the model will be the number of weekly hours/minutes dedicated to engaging in PA. The remaining variables will be evaluated to form part of the model as independent variables. The statistical analysis of the data will be conducted with SPSS software (version 26.0, IBM Corp., Armonk, NY). The treatment and analysis of the accelerometer data will be carried out with Actilife software (version 6.11.9) and R software using GGIR package. Finally, to calculate the variables of the built environment, QGIS software will be used.

## 3 Discussion

The results of the project described here will make it possible to characterize, from a scientific point of view, the environments that favor PA during pregnancy. This will provide information about possible obstetric, neonatal, and mental health benefits to pregnant women while also allowing us to analyze the associations of other potential influences of PA such as sociodemographic and psychosocial factors. With this work, our aim will be to generate the knowledge required so that specialists from different professional areas related to public health can carry out initiatives and develop government policies based on scientific criteria. This foundation will help them to implement successful interventions to improve engagement in PA by society in general, and by pregnant women in particular. In turn, this may allow pregnant women to improve their health and therefore reduce the presentation of pathologies and complications which can consequently cause greater health and social spending. In addition, because obstetric-gynecological nurses (midwives) lead the care and education of pregnant women, knowledge of their real PA levels and the determining factors for engaging in PA will help them to guide patients care more efficiently.
